# Tooth-Ache Notes

**Published:** 1899-12

**Authors:** 


### TOOTH-ACHE NOTES.

Phosphoric-acid is suitable for bleeding and swollen gums; tearing pains which are worse when warm in bed, and also from heat and from cold, burning in the front teeth during the night; pains from hollow teeth, extending into the head.

Apium-virus for the most violent pains in the gums, also for jerks and throbbing in the molars, with involuntary sudden biting together of the teeth, headache, and bleeding of the gums.

Silicea for tedious, boring, tearing pains day and night, worse during the night, spreading over the whole cheek, also into the bones of the face; discharge of offensive matter from openings near the roots of the teeth, or from the gums; swelling of the jaw.

